# Heterogeneity of Human Mast Cells With Respect to MRGPRX2 Receptor Expression and Function

**DOI:** 10.3389/fncel.2019.00299

**Published:** 2019-07-03

**Authors:** Gilda Varricchi, Antonio Pecoraro, Stefania Loffredo, Remo Poto, Felice Rivellese, Arturo Genovese, Gianni Marone, Giuseppe Spadaro

**Affiliations:** ^1^Department of Translational Medical Sciences, University of Naples Federico II, Naples, Italy; ^2^Center for Basic and Clinical Immunology Research (CISI), University of Naples Federico II, Naples, Italy; ^3^World Allergy Organization Center of Excellence, University of Naples Federico II, Naples, Italy; ^4^Center for Experimental Medicine and Rheumatology, William Harvey Research Institute, Barts and The London School of Medicine and Dentistry, Queen Mary University of London, London, United Kingdom; ^5^Institute of Experimental Endocrinology and Oncology “Gaetano Salvatore”, National Research Council (CNR), Naples, Italy

**Keywords:** heart, histamine, leukotriene C_4_, mast cells, MRGPRX2, prostaglandin D_2_, substance P and tryptase

## Abstract

Mast cells and their mediators play a role in the control of homeostasis and in the pathogenesis of several disorders. The concept of rodent mast cell heterogeneity, initially established in the mid-1960s has been extended in humans. Human mast cells isolated and purified from different anatomic sites can be activated *via* aggregation of cell surface high affinity IgE receptors (FcεRI) by antigens, superantigens, anti-IgE, and anti-FcεRI. MAS-related G protein-coupled receptor-X2 (MRGPRX2) is expressed at high level in human skin mast cells (MCs) (HSMCs), synovial MCs (HSyMCs), but not in lung MCs (HLMCs). MRGPX2 can be activated by neuropeptide substance P, several opioids, cationic drugs, and 48/80. Substance P (5 × 10^−7^ M – 5 × 10^−6^ M) induced histamine and tryptase release from HSMCs and to a lesser extent from HSyMCs, but not from HLMCs and human cardiac MCs (HHMCs). Morphine (10^−5^ M – 3 × 10^−4^ M) selectively induced histamine and tryptase release from HSMCs, but not from HLMCs and HHMCs. SP and morphine were incomplete secretagogues because they did not induce the *de novo* synthesis of arachidonic acid metabolites from human mast cells. In the same experiments anti-IgE (3 μg/ml) induced the release of histamine and tryptase and the *de novo* synthesis of prostaglandin D_2_ (PGD_2_) from HLMCs, HHMCs, HSyMCs, and HSMCs. By contrast, anti-IgE induced the production of leukotriene C_4_ (LTC_4_) from HLMCs, HHMCs, HSyMCs, but not from HSMCs. These results are compatible with the heterogeneous expression and function of MRGPRX2 receptor on primary human mast cells isolated from different anatomic sites.

## Introduction

Mast cells arise from stem cell-derived human mast cell progenitors in the bone marrow, circulate and complete their maturation in all vascularized tissues (Galli, 2016; Olivera et al., 2018). Mast cell differentiation, phenotypes and functions in tissues are largely determined by the microenvironment (e.g., cytokines, activating and inhibitory stimuli, chemokines) (Mukai et al., 2018). Mast cells are canonically implicated in allergic disorders (Liccardi et al., 2003; Fujisawa et al., 2014; Bradding and Arthur, 2016; Canonica et al., 2016; Subramanian et al., 2016; Mukai et al., 2018), but also in several non allergic conditions including autoimmune disorders (de Paulis et al., 1996; Brown and Weinberg, 2018; Rivellese et al., 2018; Yu et al., 2018), cardiovascular diseases (Patella et al., 1996, 1998; Theoharides et al., 2011; Shi et al., 2015; Ngkelo et al., 2016), bacterial (Piliponsky and Romani, 2018) and viral diseases (Haidl and Marshall, 2015), neurological disorders (Skaper et al., 2014; Theoharides et al., 2016; Skaper et al., 2017; Conti et al., 2018), and cancer (Galdiero et al., 2016; Varricchi et al., 2017). Increasing evidence supports the role of mast cells and their mediators in neurogenic inflammation leading to pain and itch (Gupta and Harvima, 2018; Steinhoff et al., 2018; Yosipovitch et al., 2018).

Enerbäck first established the concept of mast cell heterogeneity through detailed morphological and histochemical studies (Enerback, 1966a,b,c). Two distinct subpopulations of rodent mast cells, connective tissue mast cells and mucosal mast cells, differ in their location, staining features, mediator content and responsiveness to activating stimuli (Enerback, 1966a,b,c; Tainsh and Pearce, 1992; Varricchi et al., 2016). Mast cells isolated and purified from several human tissues have led to the recognition of histochemical, functional, and immunological differences among these cells in humans (Church et al., 1982; Schwartz et al., 1987; Casolaro et al., 1989; Stellato et al., 1991; Bischoff and Dahinden, 1992). For example, activation of mast cells isolated from human lung (HLMCs) by antigens, anti-IgE and superantigens leads to arachidonic acid metabolism through both the cyclooxygenase (prostaglandin D_2_, PGD_2_) and the 5-lipoxygenase pathway (peptide leukotriene C_4_, LTC_4_) (Schulman et al., 1982; de Paulis et al., 1991; Stellato et al., 1992a), whereas HSMCs only synthesize PGD_2_ (Benyon et al., 1987; Stellato et al., 1992b). Based on their protease composition, two types of human mast cells have been proposed: tryptase^+^ chymase^+^ cells (M_TC_), and tryptase^+^ chymase^−^ (M_T_), being the prototypes (Schwartz et al., 1987). However, this traditional classification is rather simplistic and mast cells show significant plasticity (Galli et al., 2011; Borriello et al., 2014). Indeed, analysis of human mast cell transcriptome demonstrated considerable greater heterogeneity across tissues than previously appreciated (Motakis et al., 2014; Dwyer et al., 2016). Moreover, recent evidence indicates that each of the two mast cell subsets originates from different precursors through several waves of mast cell differentiation, and that they display distinct surface receptors and mediators (Gentek et al., 2018; Li et al., 2018).

Human mast cells can be activated by the engagement of a plethora of receptors (Varricchi et al., 2018). Aggregation of cell surface FcεRI by antigens, anti-IgE or superantigens leads to the degranulation and the generation of newly synthesized lipid mediators, cytokines, angiogenic, and lymphangiogenic factors (Marone et al., 2006; Detoraki et al., 2009; Theoharides et al., 2010; Taracanova et al., 2018). The identification of MRGPRX2 receptor and its mouse orthologue Mrgprb2 has opened a new research avenue in mast cell biology (Tatemoto et al., 2006; Fujisawa et al., 2014; McNeil et al., 2015). MRGPRX2 can be activated by several ligands such as neuropeptides (e.g., substance P, VIP, etc.), opioids (i.e., morphine), cationic drugs (e.g., atracurium, icatibant), and 48/80 (Tatemoto et al., 2006; McNeil et al., 2015; Ali, 2017). A clinical relevance is emerging for MRGPRX2 because this receptor is implicated in drug reactions (McNeil et al., 2015) and is overexpressed in HSMCs of patients with chronic urticaria (Fujisawa et al., 2014). Gaudenzio et al. have elegantly demonstrated that substance P (SP) and IgE cross-linking (i.e., anti-IgE) induce distinct mast cell degranulation strategies in human primary MC cultures and mouse MCs (Gaudenzio et al., 2016). In this study we compared the patterns of responsiveness to anti-IgE and to MRGPRX2 agonists (SP and morphine) and the mediators synthesized by primary human lung (HLMCs), cardiac (HHMCs), skin (HSMCs), and synovial MCs (HSyMCs).

## Materials and Methods

### Reagents

HClO_4_ (Baker Chemical Co., Deventer, Netherlands), BSA, piperazine-N, N′-bis (2-ethanesulfonic acid), hyaluronidase, chymopapain, elastase type I, morphine, substance P, LTC_4_, and PGD_2_ (Sigma Chemical Co., St. Louis, MO), collagenase (Worthington Biochemical Co., Freehold, NJ), Hanks’ balanced salt solution and fetal calf serum (FCS; GIBCO, Grand Island, NY), deoxyribonuclease I and pronase (Calbiochem, La Jolla, CA), RPMI 1640 with 25 mM HEPES buffer, Eagle’s minimum essential medium (Flow Laboratories, Irvine, United Kingdom), Percoll (Pharmacia Fine Chemicals, Uppsala, Sweden), (^3^H)-LCT_4_ and (^3^H)-PGD_2_ (New England Nuclear, Boston, MA) were commercially purchased. CD117 MicroBead kit was purchased from Miltenyi Biotec (Bologna, Italy). Rabbit anti-IgE antibody was kindly donated by Dr. Kimishige Ishizaka (La Jolla Institute for Allergy and Immunology, La Jolla, CA). Rabbit anti-LTC_4_ antibody was a gift of Dr. Lawrence M. Lichtenstein (The Johns Hopkins University, Baltimore, MD). Tryptase fluoro-enzyme immune assay (FEIA; Phadia Diagnostic AB, Uppsala, Sweden) was kindly donated by Kabi Pharmacia (Milan, Italy).

### Buffers

The Pipes buffer used in these experiments was made by 25 mM Pipes, 110 mM NaCl, 5 mM KCl, pH 7.37 and referred to as P buffer. P2CG contains, in addition to P buffer, 2 mM CaCl_2_ and 1 g/l dextrose (Patella et al., 1996); pH was titrated to 7.4 with sodium bicarbonate. PGMD contains 1 mM MgCl_2_, 10 mg/l DNase, and 1 g/l gelatin in addition to P buffer, pH 7.37. The Tyrode’s buffer was made by 12 mM NaHCO_3_, 127 mM NaCl, 5 mM KCl, 0.5 mM NaH_2_PO_4_, 1 mM MgCl_2_, 5 mM glucose, and 10 mM HEPES.

### Isolation of HLMCs

The study was approved by the Ethics Committee of the University of Naples Federico II (N. 7/9). Macroscopically normal lung tissue obtained from patients undergoing lung resection for lung cancer was dissected free from pleura, bronchi, and blood vessels, minced into 5- to 10-mm fragments and dispersed into a single cell suspension as previously described (de Paulis et al., 1991; Staiano et al., 2016). Yields with this technique ranged between 0.4 × 10^5^ and 1.5 × 10^6^ mast cells per g of wet tissue and purity between 1 and 8%. Mast cells of enhanced purity (10–65%) were obtained by flotation over discontinuous Percoll gradient. Mast cells were further purified using a CD117 MicroBead kit sorting system according to the manufacturer’s instructions. Mast cell purities using this technique ranged from 85 to 98%.

### Isolation of HSMCs

Skin obtained from patients undergoing either mastectomy for breast cancer or elective cosmetic surgery was separated from the subcutaneous fat by blunt dissection. The tissue was cut into 1- to 2-mm fragments dispersed into single cell suspension as previously described (de Paulis et al., 1992). Yields with this technique ranged between 0.1 and 0.9 × 10^6^ mast cells/g of wet tissue and purities were between 4 and 8%. Mast cells were further purified using a CD117 MicroBead kit cell sorting system (Miltenyi Biotec, Bologna, Italy) according to the manufacturer’s instructions. Mast cell purities using this technique ranged from 84 to 96%.

### Isolation of HHMCs

The heart tissue used in this study was obtained from patients undergoing heart transplantation at the Deutsches Herzzentrum, Berlin, mostly for cardiomyopathy (Patella et al., 1998). The explanted heart, immediately immersed in cold (4°C) cardioplegic solution, was processed within 5–18 h of removal. Fat tissue, large vessels, and pericardium were removed. The tissue was finely minced into 2- to 5-mm fragments, suspended in P buffer (10 ml/g of wet tissue), and washed by centrifugation 3 times. After each centrifugation, the heart fragments were filtered through a 150-μm pore Nytex cloth (Tetko, Elmsford, NY). Fragments were incubated (15 min, 37°C) under constant stirring in P buffer containing 10 mg collagenase/g of wet tissue. At the end of the incubation the cell suspension was filtered through a 150-μm pore Nytex cloth and three additional cycles of enzymatic digestion were performed. After the last procedure, the cells were centrifuged (150 g, 22°C, 8 min) and filtered through a 60-μm pore Nytex cloth to remove large particles and large cells (mostly myocytes). Lastly, cells were washed twice in PGMD by centrifugation (150 g, 22°C, 8 min). Cell pellets were resuspended in P buffer containing 2% BSA and centrifuged (25 g, 22°C, 2 min) to remove sedimented myocytes. Myocytes (>100 μm long) were pelleted and discarded; supernatants containing endothelial cells, fibroblasts and mast cells were then collected and centrifuged (150 g, 22°C, 8 min). HHMCs were partially purified by flotation through a discontinuous Percoll gradient (Patella et al., 1998). The purity of these populations ranged from 0.1 to 18%. The enzymatic dispersion tissue yielded ≈ 5 × 10^4^ mast cells per gram of heart tissue. HHMCs were further purified using a CD117 MicroBead kit sorting system. Mast cell purities using these techniques ranged from 26 to 58%.

### Isolation of HSyMCs

The synovial tissue used in this study was obtained from patients with osteorthrites or rheumatoid arthritis undergoing synoviectomy. Resected joint tissue was immersed in P buffer (4°C) and was processed within 2 h of removal (de Paulis et al., 1996). Fat and fibrous tissue were removed and the tissue was finely minced into 2-5-mm fragments, suspended in P buffer (10 ml/g of wet tissue), and washed twice by centrifugation (150 g, 22°C, 8 min). The minced synovium was incubated (45 min at 37°C) with chymopapain (1 mg/ml) and pronase (0.5 mg/ml) in 1 ml Tyrode’s buffer/g synovial tissue. Remaining tissue was digested for another 45 min at 37°C with collagenase (1 mg/ml). Cell suspensions were filtered twice through 200 μ pore Nytex cloth, centrifuged (200 g, 22°C, 8 min), and washed twice with P buffer. Yields with this technique ranged from 0.2 × 10^6^ to 1.0 × 10^6^ mast cells/g of wet tissue. HSyMCs were purified by discontinuous Percoll gradient (de Paulis et al., 1996). The purity of these populations ranged from 16 to 35%. Mast cells were further purified using a CD117 MicroBead kit sorting system. HSyMCs purities using these techniques ranged from 71 to 94%.

### Histamine Release Assay

Cells (≈ 3 × 10^4^ mast cells per tube) were resuspended in P2CG, and 0.3 ml of the cell suspension were placed in 12 × 75 mm polyethylene tubes and warmed to 37°C; 0.2 ml of each prewarmed releasing stimulus (anti-IgE, substance P or morphine) was added, and incubation was continued at 37°C for 45 min (Patella et al., 1990). Cell were centrifuged (1000 g, 22°C, 2 min), and the supernatants were stored at −70°C for subsequent assay of histamine, tryptase, LTC_4_ and PGD_2_ content. Experiments with HSMCs were performed at 30°C as previously described (Stellato et al., 1992a). The cell-free supernatants were assayed for histamine with an automated fluorometric technique (Siraganian, 1974). The percent histamine release from mast cells was calculated as previously described (de Paulis et al., 1991; Varricchi et al., 2019). All values are based on means of duplicate determinations which differed by less than 10%.

### Immunoassay of Tryptase, PGD_2_, and LTC_4_

Tryptase was analyzed by FEIA (Phadia Diagnostic AB, Uppsala, Sweden) (Stellato et al., 1992a). PGD_2_ and LTC_4_ were analyzed by radioimmunoassay (Patella et al., 1990; de Paulis et al., 1991). The anti-PGD_2_ and anti-LTC_4_ antibodies had less than 1% cross-reactivity to other eicosanoids (Patella et al., 1990; de Paulis et al., 1991). Data were normalized on total cell number.

### Statistical Analysis

Values are expressed as means ± SEM. Statistical significance was assessed by using 1-way ANOVA (for data sets with Gaussian distribution) or Kruskal-Wallis test (for data sets without Gaussian distribution), followed by the Dunn multiple correction test. Results were analyzed with GraphPad Prism software (version 7.05: GraphPad Software, La Jolla, CA), and *p*-values of less than 0.05 were considered significant.

## Results

### Heterogeneous Effects of Anti-IgE on the Activation of HLMCs, HSMCs, HHMCs, and HSyMCs

**FIGURE 1:**
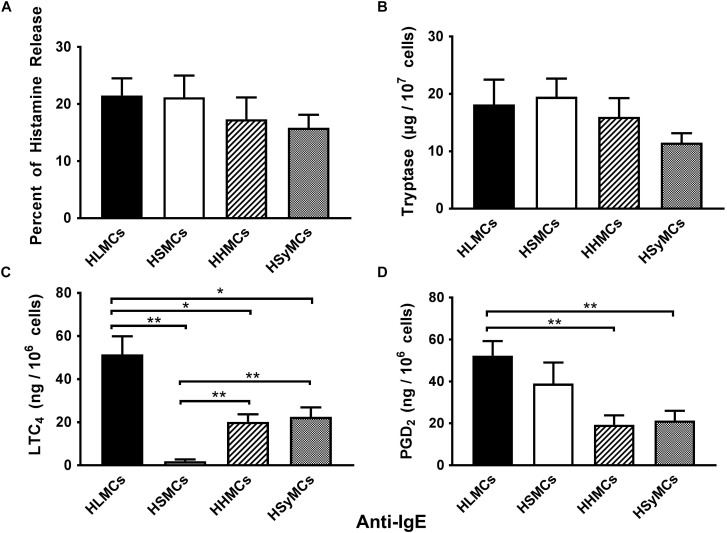
Effects of maximal stimulation of anti-IgE (3 μg/ml) on the release of histamine **(A)**, tryptase **(B)** and the *de novo* synthesis of LTC_4_ **(C)**, and PGD_2_ **(D)** from HLMCs (black bars), HSMCs (open bars), HHMCs (dashed bars), and HSyMCs (dot bars). Each point represents the mean ± SEM of six experiments in duplicate. Statistical significance was determined by ^∗^*p* < 0.05; ^∗∗^*p* < 0.01.

As previously reported (Peachell et al., 1988; de Paulis et al., 1996; Genovese et al., 2000; Varricchi et al., 2019), exposure of mast cells isolated from different anatomic sites (lung: HLMCs; skin: HSMCs; heart: HHMCs; synovial tissue: HSyMCs) to anti-IgE (10^−1^ to 3 μg/ml) resulted in a dose dependent release of histamine (data not shown). The ability of mast cells isolated from different human tissues to secrete histamine when challenged with anti-IgE indicates that they have IgE bound to FcεRI. Figure 1 summarizes the release of preformed (histamine and tryptase) and *de novo* synthesized mediators (LTC_4_ and PGD_2_) from HLMCs, HLSMCs, HHMCs, and HSyMCs when challenged anti-IgE (3 μg/ml). All types of human mast cells examined released the same percent of histamine and tryptase (Figure 1A,B). By contrast, striking differences were found among different types of mast cells when we compared the *de novo* synthesis of lipid mediators. HSMCs did not produce LTC_4_ compared to HLMCs (*p* < 0.01) and to HHMCs and HSyMCs (*p* < 0.01). Moreover, maximal stimulation of HHMCs and HSyMCs with anti-IgE led to the LTC_4_ production of 20.2 ± 3.5 and 22.5 ± 4.4 ng/10^6^ mast cells, respectively, which was significantly lower than HLMCs (51.5 ± 8.40 ng/10^6^ cells; *p* < 0.05). Interestingly, the anti-IgE-mediated production of PGD_2_ from HLMCs (52.3 ± 6.9 ng/10^6^ mast cells) and HSMCs (39.0 ± 10.0 ng/10^6^ mast cells) did not differ between the two groups. However, only the production of PGD_2_ from HLMCs, but not HSMCs, was significantly higher than that produced by HHMCs (19.3 ± 4.5 ng/10^6^ mast cells) and HSyMCs (21.3 ± 4.6 ng/10^6^ mast cells) (*p* < 0.01). Collectively these results identify striking differences with respect to the release of different types of mediators in response to IgE-mediated stimuli among human mast cells isolated from different anatomic sites.

### Heterogeneous Effects of Substance P on the Activation of HLMCs, HSMCs, HHMCs, and HSyMCs

**FIGURE 2:**
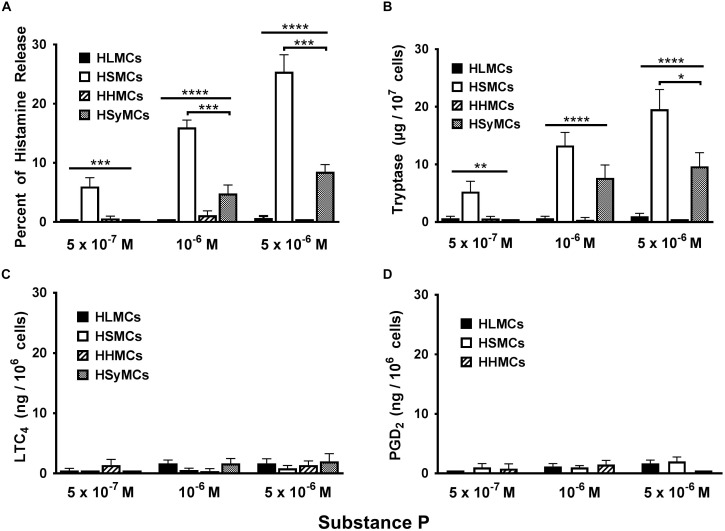
Effects of increasing concentrations of substance P (5 × 10^−7^ M to 5 × 10^−6^ M) on the release of histamine **(A)**, tryptase **(B)**, and the *de novo* synthesis of LTC_4_ **(C)**, and PGD_2_ **(D)** from HLMCs, HSMCs, HHMCs, and HSyMCs. Each point represents the mean ± SEM of six experiments in duplicate. Statistical significance was determined by ^∗^*p* < 0.05; ^∗∗^*p* < 0.01; ^∗∗∗^*p* < 0.001; ^∗∗∗∗^*p* < 0.0001.

Substance P (SP) has long been established as an inflammatory neuropeptide (O’Connor et al., 2004; Mashaghi et al., 2016) and potent endogenous pruritogen in mice and humans (Azimi et al., 2017; Gupta and Harvima, 2018; Yosipovitch et al., 2018). Although the classical receptor for SP is the neurokinin-1 receptor (NK-1R) (Douglas and Leeman, 2011), recent studies have demonstrated that SP activates MRGPRX2 receptor in addition to NK-1R to induce itch (Azimi et al., 2017). There is also evidence that SP can activate adventitial mast cells (Bot et al., 2010). Moreover, SP can be released into joint tissues from sensory nerve fibers (Pereira da Silva and Carmo-Fonseca, 1990; Gronblad et al., 1991) and its concentrations are increased in synovial fluid from patients with rheumatoid arthritis (Devillier et al., 1986). We therefore compared the effects of increasing concentrations (5 × 10^−7^ to 5 × 10^−6^ M) of SP on the activation of HLMCs, HSMCs, HHMCs, and HSyMCs. Figure 2 shows that SP caused concentration-dependent histamine and tryptase release from HSMCs whereas it had no effect on both HLMCs and HHMCs. SP caused histamine and tryptase release from HSyMCs only at the higher concentrations (10^−6^ M and 5 × 10^−6^ M) examined. The percent histamine release from HSyMCs caused by the latter concentrations of SP was significantly lower (*p* < 0.001) than that induced from HSMCs. Interestingly, in these experiments SP did not induce the metabolism of arachidonic acid through the 5-lipoxygenase pathway (LTC_4_) (Figure 2C) and the cyclooxygenase (PGD_2_) (Figure 2D) in all types of mast cell examined.

### Heterogeneous Effects of Morphine on the Activation of HLMCs, HSMCs, HHMCs, and HSyMCs

**FIGURE 3:**
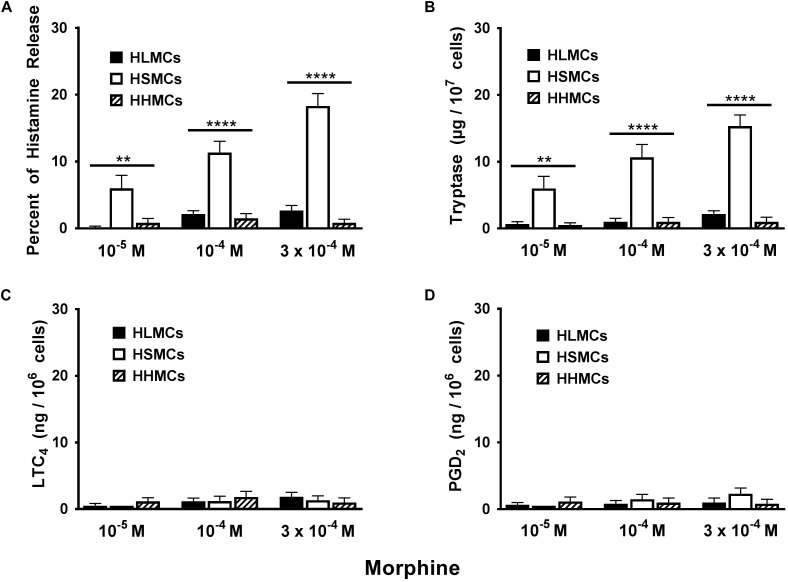
Effects of increasing concentrations of morphine (10^−5^ M to 3 × 10^−4^ M) on the release of histamine **(A)**, tryptase **(B)** and the *de novo* synthesis of LTC_4_ **(C)**, and PGD_2_ **(D)** from HLMCs, HSMCs and HHMCs. Each point represents the mean ± SEM of six experiments in duplicate. Statistical significance was determined by ^∗∗^*p* < 0.01; ^∗∗∗∗^*p* < 0.0001.

Opioid compounds bind to multiple receptors also present on several cells of innate and adaptive immunity where they exert immunomodulatory effects (Boland and Pockley, 2018; Plein and Rittner, 2018). Recent evidence indicates that several opioid compounds including morphine can activate the human LAD2 mast cell line through MRGPRX2 (Lansu et al., 2017). Figure 3 shows the results of several experiments comparing the effects of increasing concentrations (10^−5^ to 3 × 10^−4^ M) of morphine on mediator release from primary HLMCs, HSMCs, and HHMCs. Morphine selectively induced histamine and tryptase release from HSMCs but not from HLMCs and HHMCs. Interestingly, morphine was an incomplete secretagogue because it did not induce the production of both LTC_4_ and PGD_2_ from all types of human mast cells.

## Discussion

The results of this study extends previous findings demonstrating the functional heterogeneity of human primary mast cells isolated from different anatomic sites with respect to FcεRI-mediated activation (Schwartz et al., 1987; Casolaro et al., 1989; Stellato et al., 1992a; Patella et al., 1998; Galli et al., 2011; Motakis et al., 2014). No differences were found with respect to anti-IgE-mediated release of preformed mediators (histamine and tryptase) from human primary mast cells purified from different anatomic sites. By contrast, striking differences were demonstrated among different types of mast cells with respect to the anti-IgE-induced *de novo* synthesis of LTC_4_ and PGD_2_. Interestingly, LTC_4_ is not produced by anti-IgE-activated HSMCs whereas these cells synthesize PGD_2_. Moreover, the IgE-mediated production of both LTC_4_ and PGD_2_ from HLMCs was higher than that of HHMCs and HSyMCs. Collectively, these results suggest the existence of profound differences between the biochemical mechanisms that regulate the secretion of preformed mediators and the *de novo* production of lipid molecules among different types of human primary mast cells.

Tatemoto and coworkers first demonstrated the presence of MRGPRX2 mRNA in human skin and in human cord blood mast cells (CBMCs) (Tatemoto et al., 2006). They also found that several basic secretagogues, including SP, induced mast cell degranulation. They suggested that MRGPRX2 receptor is highly expressed in MC_TC_ compared to M_T_. Since this initial observation several groups have demonstrated that various neuropeptides (e.g., VIP), endogenous and exogenous opioids (e.g., morphine), cationic drugs (e.g., icatibant, atracurium, ciprofloxacin), and 48/80 can activate human mast cells via the MRGPRX2 receptor (McNeil et al., 2015; Gaudenzio et al., 2016; Lansu et al., 2017; Okamura et al., 2017; Alkanfari et al., 2018). Interestingly the group of Theoharides has demonstrated that SP can synergistically potentiate the production of several cytokines (e.g., TNF-α, VEGF, IL-1β) by LAD2 mast cells (Theoharides et al., 2010; Taracanova et al., 2017, 2018).

The activating property of MRGPRX2 agonists has been evaluated in human LAD2 cell line (Guhl et al., 2005; Kulka et al., 2008; Theoharides et al., 2010; McNeil et al., 2015), human peripheral blood-derived cultured mast cells (PBCMCs) (Gaudenzio et al., 2016), and human CBMCs (Tatemoto et al., 2006). In the present study performed using primary mast cell isolated and purified from different human tissues, we found that two MRGPRX2 agonists, SP and morphine, selectively induce the release of preformed mediators (histamine and tryptase) from HSMCs, but not from HLMCs and HHMCs. These findings are consistent with the observation that the MRGPRX2 receptor is expressed in HSMCs but not in lung mast cells (Fujisawa et al., 2014; Babina et al., 2018). We also found that high concentrations of SP caused small but significant release of histamine and tryptase from HSyMCs. This observation could be of some interest because Okamura et al. have demonstrated that SP activates HSyMCs to release histamine and to produce PGD_2_ (Lee et al., 2013) through the activation of MRGPRX2 (Okamura et al., 2017). In our study SP caused some release of preformed mediators (i.e., histamine and tryptase) from HSyMCs, but not the *de novo* synthesis of both PGD_2_ and LTC_4_. Several studies have suggested the involvement of SP in experimental arthritis (Levine et al., 1984; Ahmed et al., 1995; Seegers et al., 2003) and in rheumatoid arthritis (Hernanz et al., 1993; Menkes et al., 1993; Miller et al., 2000; Grimsholm et al., 2005; Dirmeier et al., 2008). Further studies are needed to clarify the SP-mediated production of proinflammatory and immunomodulatory mediators from HSyMCs.

There is increasing evidence that cardiac mast cells play a role in several myocardial disorders (Patella et al., 1990, 1996, 1998; Theoharides et al., 2011; Shi et al., 2015; Ngkelo et al., 2016; Marino et al., 2017). It has been reported that SP induces adverse myocardial remodeling (Melendez et al., 2011) and intraplaque hemorrhage in atherosclerosis (Bot et al., 2010) *via* the activation of mast cells. Azimi and collaborators have implicated mast cell MRGPRX2 in human and experimental cardiometabolic disorders (Azimi et al., 2017). However, the mechanism(s) of SP-mediated vascular and cardiac mast cell activation remains controversial (Shi et al., 2017). In our study SP and morphine failed to induce the release of preformed and *de novo* synthesized mediators from partially purified HHMCs. Interestingly, we have previously demonstrated by immunoelectron microscopy the presence of both tryptase and chymase in human cardiac mast cells (Patella et al., 1995). Thus, although HHMCs contain both serine proteases, similarly to HSMCs, they differ from the latter in response to MRGPRX2 activators. Several explanations can justify this intriguing observation: first, the possibility of the existence of MRGPRX2 variants expressed in different types of human mast cells (Alkanfari et al., 2018) cannot be excluded; second, the complex enzymatic and mechanical procedure to purify HHMCs might alter the expression and function of MRGPRX2 both at the plasma membrane and intracellular sites (Fujisawa et al., 2014). We are presently investigating the surface and intracellular localization of MRGPRX2 in HHMCs to explain the apparent lack of functional effects of SP and morphine on these cells.

Increasing evidence supports the role of mast cells in neurogenic inflammation (Skaper et al., 2014; Skaper et al., 2017) leading to itch and pain (Vincent et al., 2013; Gupta and Harvima, 2018; Yosipovitch et al., 2018). Nerve fibers release proinflammatory and vasoactive neuropeptides such as SP (Rosa and Fantozzi, 2013; Skaper et al., 2017), which can activate mast cells. These cells release algogenic and pruritogenic mediators such as tryptase and histamine (Yosipovitch et al., 2018), which activate specific nociceptors on sensory nerve fibers (Vergnolle et al., 2001; Rosa and Fantozzi, 2013). There is increasing evidence that SP is linked to itch and pain through activation of MRGPRX2 on mast cells and sensory neurons (Azimi et al., 2016, 2017). We found that SP is a potent activator of the release of both histamine and tryptase from HSMCs that highly express MRGPRX2 (Fujisawa et al., 2014). The role of tryptase is particularly relevant because this protease activates the PAR2 receptor on nerve endings (Vergnolle et al., 2001; Zhang et al., 2012) stimulating the release of SP and other neuropeptides (Steinhoff et al., 2000) that activate nociceptors on nerve terminals as well as mast cells in a paracrine manner. Moreover, *in vivo* administration of morphine can induce histamine release (Baldo and Pham, 2012; Kumar and Singh, 2013) and itching in humans presumably *via* MRGPRX2-mediated HSMC activation.

Our study has some limitations which have to be pointed out. It was performed using primary mast cells isolated from several tissues (i.e., lung, heart, synovial, skin) obtained from different patients. Moreover, these mast cells might have different characteristics from cells obtained from healthy donors. Finally, the mechanical and enzymatic procedures to isolate mast cells from different anatomic sites are quite different. We cannot exclude the possibility that the techniques used to isolate and purify mast cells from different tissues might explain, at least in part, their different response to MRGPRX2 activation.

In conclusion, the results of this study demonstrate that there is greater functional heterogeneity of primary human mast cells across tissues than previously appreciated. First, we extend previous findings demonstrating heterogeneity when different types of human mast cells are activated *via* aggregation of FcεRI by anti-IgE. Second, there is heterogeneity of *de novo* synthesized mediators produced by different human mast cells activated by IgE-cross-linking. Third, there is heterogeneity of human mast cells with respect to MRGPRX2 activation. Additional studies are needed to examine the intracellular and membrane expression of MRGPRX2 in different types of primary human mast cells.

## Data Availability

The datasets for this study will not be made publicly available because Some of the data are part of a patent.

## Author Contributions

GV, AG, SL, RP, GM, and GS have conceived and designed the study. GV, AP, SL, RP, FR, and GM performed the experiments. AP performed the statistical analysis of the results. AP and GM elaborated the figures. All the authors contributed intellectually and to the writing of the final version of the manuscript.

## Conflict of Interest Statement

The authors declare that the research was conducted in the absence of any commercial or financial relationships that could be construed as a potential conflict of interest.
